# A review of the effectiveness of stress management skills training on academic vitality and psychological well-being of college students

**Published:** 2015

**Authors:** P Alborzkouh, M Nabati, M Zainali, Y Abed, F Shahgholy Ghahfarokhi

**Affiliations:** * Exceptional Children Psychology, Islamic Azad University, Central Tehran Branch, Iran; ** General Psychology, Islamic Azad University, South Tehran Branch, Iran; *** General Psychology, Humanities and Social Sciences Faculty, Paradise University, Gillan Branch, Iran; **** General Psychology, Islamic Azad University, Science and Research Branch, Tehran, Iran; ***** Clinical Psychology, Islamic Azad University, Science and Research Branch Branch, Isfahan, Iran

**Keywords:** stress management skills training, psychological well-being, academic vitality, students

## Abstract

**Objective:** Carrying out the appropriate psychological interventions to improve vitality and mental well-being is critical. The study was carried out to review the effectiveness of stress management training on the academic life and mental well-being of the students of Shahed University.

**Methodology:** The method used was quasi-experimental with a pretest-posttest plan and control group. Therefore, a total of 40 students of Shahed University of Tehran were selected by a convenience sampling method and were organized into two groups: experimental and control group. Both groups were pretested by using an academic vitality inventory and an 84-question psychological well-being inventory. Then, the experimental group received stress management skills training for ten sessions, and the control group did not receive any intervention. Next, both groups were post-tested, and the data were analyzed with SPSS-21 software by using descriptive and inferential statistical methods.

**Findings:** The findings showed that the stress management skills training significantly contributed to promoting the academic vitality and psychological well-being of students (p < 0.001).

**Conclusions:** It was concluded from this research that teaching the methods for dealing with stress was an effective strategy to help students exposed to high stress and pressure, and this was due to its high efficiency, especially when it was held in groups, had a small cost, and it was accepted by the individuals.

## Introduction

Challenges during education create sources of stress for students, and put their health at risk, in a way that affects their learning abilities [**1**]. Therefore, paying attention to the factors that could have a positive impact on the agreeableness and could increase the positive psychological states, and as a result, the physical and psychological health of the students was of great importance. 

Among the important factors that affect people’s ability to adapt to the stresses of studying era is academic vitality [**2**]. Academic vitality means an adaptive response to various challenges and barriers experienced during education [**3**]. When a person does things spontaneously, does not feel not only frustrated and tired, but also constantly feels the strength and increased energy, and overall has a sense of inner vitality [**2**]. Therefore, the academic life has a relationship with the individual’s adaptation to the various situations of the academic period, feelings of self-efficacy and empowerment in the face of challenges, experiencing less anxiety and depression, a sense of responsibility in dealing with the academic tasks and better academic success [**3**]. Despite the high importance of academic vitality in the successful confrontation with the challenging academic period, the literature review of the studies managed in Iran showed that few studies were performed on the factors promoting this important variable. Therefore, an attempt to address this research gap increased the need for the current study. 

Another important positive psychological state in students is the psychological well-being. The psychological well-being factor is defined as a person’s real talents growth and has six components that are the purpose in life, positive relations with others, personal growth, self-acceptance, autonomy, and environmental mastery [**4**]. The purpose in life means having a purpose and direction in life and pursuing them [**5**]. Positive relations with the others mean having warm, satisfactory relations along with confidence and empathy [**6**]. Personal growth means having a sense of continuous growth and the capacity for it and having an increased sense of efficacy and wisdom [**4**]. Self-acceptance means having a positive attitude towards oneself and accepting the various aspects of oneself [**6**]. Autonomy means the feeling of self-determination, independence, and self-assessment against personal criteria [**4**]. Moreover, environmental mastery means a sense of competence and the ability to manage the complex environment around [**5**]. 

However, one of the most significant parts affecting the psychological health and well-being of individuals is life skills training [**7**]. Life skills’ training is critical for students, in a way that on this basis, many universities have started to teach life skills and stress management skills to improve the physical and psychological health of their students in the recent years [**8**]. The main objective of the World Health Organization regarding the creation of a life skills plan is in the field of psychological health. Therefore, different societies throughout the world try to promote the implementation and evaluation of the programs training in life skills. It focuses on the growth of mental abilities such as problem-solving, coping with emotions, self-awareness, social harmony, and stress management among children, teenagers, and even adults [**9**]. From the life skills, training in stress management skills is critical, because students need to deal effectively with stressful issues and factors. Accordingly, it was thought that teaching stress management skills is very efficient in improving the students’ positive psychological states, in particular, their vitality and mental well-being. Therefore, this study examined the effectiveness of the stress management skills training on the academic life and psychological well-being among Shahed University students.

## Methodology

The study was quasi-experimental with a pretest-posttest. The analytical community of the study included all the students of Shahed University of Tehran in the fall of 2015, who were selected with a convenience method. For the calculation of the sample size, the appropriate sample size in experimental studies was of 15 people for each group [**10**]. At first, the sample size of 15 individuals was selected for each group. Then, to increase the statistical power and to manage the possible decrease in the number of participants, the sample size of 20 individuals (n = 20) was considered for each group. The sampling was voluntary non-random from among all the students studying at Shahed University. The inclusion criteria included an informed consent and the willingness to participate in the research, the ability to take part in the sessions and to collaborate in carrying out assignments, willingness to cooperate in completing the instruments, and the age range of 18 to 35 years. The exclusion criteria included the lack of desire to participate in the sessions and the absence to more than three courses in the preparation method, the lack of the ability to participate in the sessions, lack of cooperation in carrying out assignments, and receiving any training or psychological therapy that was not part of the program of this research. 

The procedure of the study was that from all the students studying at Shahed University, a number was non-randomly and voluntarily selected, and if they met the inclusion criteria, they were randomly assigned to two groups: experimental and control. At the beginning and before starting the study, an informed consent was obtained from all of them to uphold moral considerations, through informing them of the aim of the study and the impact of such studies in improving their psychological status. Then, all the information of the participants were collected, and they were assured that the information would remain confidential by the researcher. Then, the experimental group received group stress management training for ten sessions, and the control group did not receive any intervention. In the end, both groups were post-tested. The protocol of stress management training sessions is presented in **Table 1**.

The instruments used in the study included a demographic sample page, an academic vitality questionnaire, and a psychological well-being scale (PWBS-18).

Demographic sample page: The demographic sample page included age, gender, educational level, and marital status. The sample page was prepared and evaluated by the researchers of the study. 

Academic vitality questionnaire: This questionnaire was developed by Dehqanizadeh MH, Hosseinchari M (2012) [**3**], based on the academic vitality scale of Martin AJ, Marsh HW (2006) [**15**], which had four items. After various implementations of the items of the questionnaire, the final version was rewritten, and the result was that the revised version had ten items. Then the items above were again examined in a preliminary study on a sample including 186 high school students, who were chosen by using a cluster random sampling, and their psychometric properties were examined. The results of the examination showed that the obtained Cronbach’s alpha coefficient, by removing [**3**] item number 8, was 0.80 and the retest coefficient was 0.73. Also, the range of correlation of the elements with the total score was between 0.51 and 0.68. These results indicated that the items had a satisfactory internal consistency and stability. 

Psychological well-being scale (SPWB): Riffe’s mental well-being scale [**11**] was made up of 84 questions in Likert’s 7-degree scale (from “strongly disagree” to “agree strongly”). It was a self-report questionnaire, which measured six components of the psychological well-being, including purpose in life, positive relations with others, personal growth, self-acceptance, autonomy, and environmental mastery. The internal consistency coefficients for the components of this questionnaire were obtained from 0.83 to 0.91. In Mohammadpour and Joshanloo research (2014) [**6**], the reliability coefficient of this scale with Cronbach’s alpha method for the psychological well-being scale obtained was 0.81. Also, for the subscales of the test including self-compliance, environmental mastery, personal growth and development, link with others, the goal in life, and self-acceptance were obtained at 0.60, 0.64, 0.54, 0.58, 0.65, and 0.61, respectively. A study performed by Kafka and Kozma (2002) was conducted to verify the validity of the items of the Riffe’s psychological well-being scale. The findings showed that there was a high correlation between this scale and the subjective well-being scale (SWB) and the satisfaction with life scale (SWLS). In the present study, the reliability coefficient with Cronbach’s alpha method for the psychological well-being scale obtained was 0.81. Also, for the subscales of the test, including self-compliance, environmental mastery, personal growth and development, relations with others, the goal in life, and self-acceptance were obtained at 0.60, 0.64, 0.54, 0.58, 0.65, and 0.61, respectively.

The SPSS-20 software was used for data analysis. The statistical method used for the data analysis of the research on the level of descriptive statistics was mean, standard deviation, frequency, and frequency percentage indexes, and on the inferential statistics, univariate and multivariate analysis of covariance model were used.

**Table 1:** Protocol of stress management skills training sessions

Session	Subject
First	Acquaintance of group members, practicing acquaintance, introducing stress, stress creating factors and responses to stress, and getting to know the physical effects of stress
Second	Raising awareness of the effects of stress and understanding the importance of this awareness and increasing awareness of the physical responses related to stress creating factors
Third	Explaining the relationship between thoughts, emotions, and physical senses, and providing numerous examples in different positions
Fourth	Introducing and identifying the common types of negative thoughts and cognitive distortions
Fifth	Challenging the common negative thoughts and cognitive distortions and replacing irrational thoughts with rational ones
Sixth	Instruction, practicing, and implementing effective coping strategies
Seventh	Continuing the training, practicing, and implementing of effective coping strategies
Eighth	Training and discussion about anger management, assertiveness, time management, and recording daily events
Ninth	Learning to use problem-solving skills in conflicts, discussing about the skills of saying “No”, and delegating authority
Tenth	Learning the importance and understanding the benefits of social protection and an overview of the program

**Findings of the research**

The demographic properties of the sample present in the study are presented in **Table 2**.

**Table 2:** Demographic characteristics of the subjects

Variable	Group	Frequency	Frequency percentage	Mean and standard deviation
Age	18 to 20 years	6	15	24.85 ± 4.41
	21 to 25 years	14	35	
	26 to 30 years	11	27.5	
	31 to 35 years	9	22.5	
Education	Bachelor’s degree	33	82.5	
	Master’s degree	7	17.5	
Marital status	Single	37	92.5	
	Married	3	7.5	

As presented in **Table 1**, the largest frequency of participation belonged to the participants in the age range of 21 to 25 with 14 individuals (35%) and the lowest frequency of individuals in the range of 18 to 20 years, with six individuals (15%). In addition, the mean age of the participants was 24.85, and the standard deviation was 4.41. The other information about the demographic properties of the present sample is provided in **Table 2**

**Table 3:** Descriptive stats of academic vitality and psychological well-being scores of the two groups divided by the pretest and posttest

Component	Index	Experimental		Control	
		Pretest	Posttest	Pretest	Posttest
Purpose in life	Mean	49.50	59.10	50.55	50.15
	Standard deviation	6.38	7.67	5.08	4.86
Positive relations with others	Mean	52.25	61.95	50.05	50.31
	Standard deviation	5.58	7.07	3.28	3.97
Personal growth	Mean	52.50	60.25	51.80	51.25
	Standard deviation	3.85	4.23	2.54	2.44
Self-acceptance	Mean	48.94	60.20	49.45	49.67
	Standard deviation	3.15	3.83	4.17	4.12
Autonomy	Mean	47.45	57.50	49.50	48.60
	Standard deviation	2.91	4.21	3.99	3.26
Environmental mastery	Mean	48.15	58.30	47.45	47.40
	Standard deviation	5.15	4.06	5.06	5.45
Psychological well-being	Mean	298.80	357.30	296.95	297.30
	Standard deviation	12.85	14.15	11.40	11.76
Academic vitality	Mean	21.50	32.20	21.95	22.80
	Standard deviation	4.28	5.26	3.41	3.33

As shown in **Table 3**, the mean scores of purpose in life, positive relations with others, personal growth, self-acceptance, autonomy, environmental mastery, total score of psychological well-being, and academic vitality of posttest were increased in the test group as associated with the control group. 

**Table 4:** Results of Levene test for the examination of the consistency of variances of academic vitality and psychological well-being variables with its components in the posttest stage

Variable	Stage	F	Degree of freedom 1	Degree of freedom 2	Significance level
Purpose in life	Posttest	2.265	1	38	0.141
Positive relations with others	Posttest	0.201	22	1	1.734
Personal growth	Posttest	0.622	22	1	0.251
Self-acceptance	Posttest	0.054	1	38	0.817
Autonomy	Posttest	2.091	1	38	0.156
Environmental mastery	Posttest	1.458	1	38	0.235
Psychological well-being	Posttest	0.049	1	38	0.826
Academic vitality	Posttest	2.331	1	38	0.135

As shown in **Table 4**, the null hypothesis of the equality of variances of the two groups in the academic vitality and psychological well-being with all its components was confirmed. It meant that the variances of the two clusters in the population were equal and had no significant difference for the academic vitality and the psychological well-being variable with all its components. Thus, given the compliance with the Levene assumption, the analysis of covariance of the results of the hypothesis of the research were permitted. 

**Table 5:** Results of multivariate analysis of covariance on the scores of posttest with the control of pretest in the academic vitality and psychological well-being variable with its components

Test name	Value	F	Degree of freedom	Significance level	Eta square	Power
Pyllai’s trace	0.896	47.249	6	0.001	0.896	0.95
Wilkes’s lambda	0.104	47.249	6	0.001	0.896	0.95
Hotelling’s trace	8.591	47.249	6	0.001	0.896	0.95
Roy’s largest root	8.591	47.249	6	0.001	0.896	0.95

As shown in **Table 5**, the significance level of all the tests (p < 0.001) indicated that there was a significant difference between the two groups at least in one of the dependent variables (academic vitality and psychological well-being with its components). And, according to the eta square, 0.89 percent of the differences observed among individuals were associated with the effect of the independent variable, which was the intervention method (stress management skills training). On the other hand, given that the statistical power was 0.95, which was higher than 0.80, the sample size was acceptable for the research. The results related to significant differences in any of the dependent variables are listed below. 

**Table 6:** The results of multivariate analysis of covariance to assess the impact of stress management skills training on the level of psychological well-being and its components in the posttest stage

Index	Sum of squares	Degree of freedom	Mean squares	F	Significance level	Eta square
Academic vitality	883.601	1	883.601	45.472	0.001	0.545
Purpose in life	810.003	1	810.003	19.585	0.001	0.341
Positive relations with others	1357.225	1	1357.225	42.097	0.001	0.526
Personal growth	810.001	1	810.001	67.574	0.001	0.640
Self-acceptance	1113.025	1	1113.025	70.287	0.001	0.649
Autonomy	792.100	1	792.100	55.761	0.001	0.595
Environmental mastery	1188.100	1	1188.100	51.363	0.001	0.575
Psychological well-being	36007.001	1	36007.001	212.607	0.001	0.848

According to **Table 6**, the significance level was p < 0.001, the hypothesis of the difference between the academic vitality and the psychological well-being with its components in the two groups was confirmed. It stated that 0.54, 0.25, 0.52, 0.64, 0.60, 0.59, 0.45 and 0.81 percent change in the academic vitality, individuals’ purpose in life, positive relations with others, personal growth, self-acceptance, autonomy, environmental mastery, and psychological well-being scores were due to the independent variable (stress management skills training). Therefore, it could be said that stress management skills training increased the academic vitality and the psychological well-being and all of its components. 

## Discussion and conclusions

Given the aim of this study, which was to examine the effectiveness of stress management skills training on the academic vitality and psychological well-being of the students of Shahed University, the results of the univariate and multivariate analysis of covariance showed that stress management skills training had a significant impact on increasing the academic vitality and psychological well-being. The findings indicated that the stress management skills training had a major impact on increasing the academic life. It was consistent with different studies of Habibi M (2015), Pakdaman A, Ganji K, Ahmadzadeh M (2012), Shirbim Z, Sudani M, Shafi-Abadi A (2008) [**12**-**14**].

In explaining their similar finding, Pakdaman A, Ganji K, Ahmadzadeh M (2012) [**13**] also stated that life skills training helped in the improvement of the academic conditions of the subjects. In addition, this was because of this training, with growing different skills of the students, helping the students know their strengths and weaknesses, and overall, help the individuals move from weaknesses and skill deficits to capable and strong skills. Therefore, this could provide the students with better educational conditions [**14**]. In explaining their similar finding, Shafi-Abadi (2008) stated that teaching life skills, including stress management skills, are one of the ways to improve the mental health of the individuals of the community and to prevent harms. In fact, these teachings protected the health and mental hygiene of the society and protected it against diseases, disabilities, and disturbances in human relations. As a result, the feeling of security and solidarity increased among the members of the society, and then their senses of happiness, vitality, and health increased.

The findings showed that stress management skills’ training has a significant impact on the psychological well-being. It was consistent with the multiple studies of Qadiri-Bahramabadi F, Mikaeli-Manee F (2015), Qanbari N, Habibi M, Shams-Aldini S (2013), Alavi-Arjmand N, Kashaninia Z, Hosseini MA, Reza-Soltani P (2012), Chubforushzadeh A, Kalantari M, Molavi H (2009) [**16**-**19**]. 

In explaining their similar findings, Qadiri-Bahramabadi F, Mikaeli-Manee F (2015) [**16**] stated that facing numerous stresses required teaching and learning of appropriate stress management skills. In other words, during stress, individuals must know the necessary coping skills to reduce the effects of stress, and if the pressure was managed and the effective coping skills were applied, the person would be able to get along better with the needs and challenges of his/ her life. Therefore, the intervention of stress management led to the formation of good feelings about oneself, as well as a positive performance in the stable world. It created interest and motivation in people’s lives as well as increasing the self-confidence of the individuals. As a result, it increased the psychological well-being. 

In explaining their similar finding, Qanbari N, Habibi M, Shams-Aldini S (2013) [**17**] stated that with the help of multiple strategies to manage stress such as relaxation, and muscular relaxation, stress and anxiety could be reduced. The individuals identified the somatic symptoms, and with mastering the ways to acquire relaxation, which was inconsistent with stress, reduced their anxiety and unpleasant feelings, thus increasing the psychological well-being. Also, in explaining their similar finding, Chubforushzadeh A, Kalantari M, Molavi H (2009) [**19**], stated that stress management treatments make multiple changes in the individual’s beliefs, feelings, and behaviors. Therefore, improving the individual’s evaluations and coping skills, and the provided practices to integrate the learned separations with real life situations could lead to a decrease in the perceived stress and an increase in the psychological well-being.

**Acknowledgement**

The authors would like to thank the venerable authorities of Shahed University of Tehran for their assistance. Also, the authors would like to thank all the participants in the study.
